# Assessing Autophagy in Muscle Stem Cells

**DOI:** 10.3389/fcell.2020.620409

**Published:** 2021-01-21

**Authors:** Silvia Campanario, Ignacio Ramírez-Pardo, Xiaotong Hong, Joan Isern, Pura Muñoz-Cánoves

**Affiliations:** ^1^Centro Nacional de Investigaciones Cardiovasculares (CNIC), Madrid, Spain; ^2^Department of Experimental and Health Sciences, Pompeu Fabra University (UPF), CIBER on Neurodegenerative Diseases (CIBERNED), Barcelona, Spain; ^3^ICREA, Barcelona, Spain

**Keywords:** autophagy, stem cell, satellite cell, skeletal muscle, regeneration, quiescence, flow cytometry, immunofluorescence

## Abstract

The skeletal muscle tissue in the adult is relatively stable under normal conditions but retains a striking ability to regenerate by its resident stem cells (satellite cells). Satellite cells exist in a quiescent (G0) state; however, in response to an injury, they reenter the cell cycle and start proliferating to provide sufficient progeny to form new myofibers or undergo self-renewal and returning to quiescence. Maintenance of satellite cell quiescence and entry of satellite cells into the activation state requires autophagy, a fundamental degradative and recycling process that preserves cellular proteostasis. With aging, satellite cell regenerative capacity declines, correlating with loss of autophagy. Enhancing autophagy in aged satellite cells restores their regenerative functions, underscoring this proteostatic activity’s relevance for tissue regeneration. Here we describe two strategies for assessing autophagic activity in satellite cells from GFP-LC3 reporter mice, which allows direct autophagosome labeling, or from non-transgenic (wild-type) mice, where autophagosomes can be immunostained. Treatment of GFP-LC3 or WT satellite cells with compounds that interfere with autophagosome-lysosome fusion enables measurement of autophagic activity by flow cytometry and immunofluorescence. Thus, the methods presented permit a relatively rapid assessment of autophagy in stem cells from skeletal muscle in homeostasis and in different pathological scenarios such as regeneration, aging or disease.

## Introduction

**FIGURE 1:**
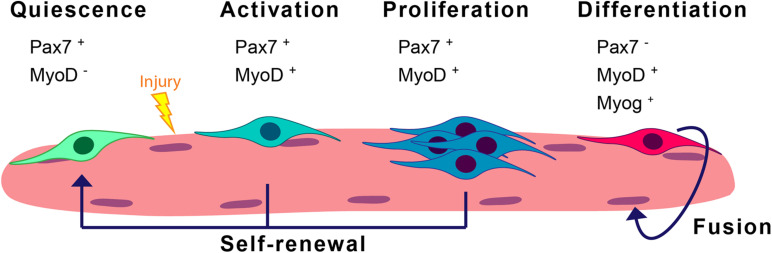
Scheme of the satellite cell myogenic program. SCs are normally in quiescence and enter a myogenic cycle upon stress conditions such as injury. In the steady-state, they express Pax7. Activation of SCs can be determined by the co-expression of Pax7 and the myogenic regulatory factor MyoD. Proliferating SCs later differentiate into differentiated/committed progenitors characterized by the downregulation of Pax7 expression and the induction of Myog expression. These differentiated cells will eventually fuse into myofibers. A subset of the proliferating SCs will return to quiescence through the process of self-renewal.

Skeletal muscle is formed by multinucleated myofibers and exhibits a remarkable capacity to regenerate thanks to its resident stem cells, also called satellite cells (SCs) (Mauro, 1961). These cells are characterized by the expression of the paired-box transcription factor Pax7 (Seale et al., 2004), and constitute the main source of new myonuclei for myofiber growth and regeneration. In homeostasis, SCs are in a reversible G_0_ arrest state called quiescence and present low transcriptional and metabolic activities. In response to muscle injury, SCs activate and orchestrate a myogenic program to regenerate the damaged muscle (see detailed myogenic states and markers in Figure 1). Activated SCs rapidly proliferate, and differentiate and fuse to form new regenerating myofibers and reconstitute the muscle tissue. Alternatively, SCs undergo self-renewal to replenish the quiescent stem-cell pool (revised in Evano and Tajbakhsh, 2018; Feige et al., 2018). SC regenerative functions decline with aging, and this decline is maximal at geriatric age (Sousa-Victor et al., 2014). Likewise, SC functions are altered in muscle diseases such as in the severe Duchene muscular dystrophy (DMD) (Dumont et al., 2015; Chang et al., 2018). Maintenance of the SC quiescent state needs basal surveillance mechanisms to maintain the cell’s proteome quality and overall homeostasis. The entrance of SCs into an activated state in response to local muscle damage requires rapid protein composition changes, eliminating proteins involved in maintaining the quiescent state and supplying new proteins involved in cell-cycle regulation and differentiation. In particular, SC quiescence and activation after injury both require macroautophagy (Tang and Rando, 2014; García-Prat et al., 2016). Macroautophagy (hereafter called autophagy) is a regulated recycling mechanism that dismantles unnecessary or dysfunctional cell components, ranging from small macromolecules to full-sized organelles (Mizushima and Komatsu, 2011).

**FIGURE 2:**
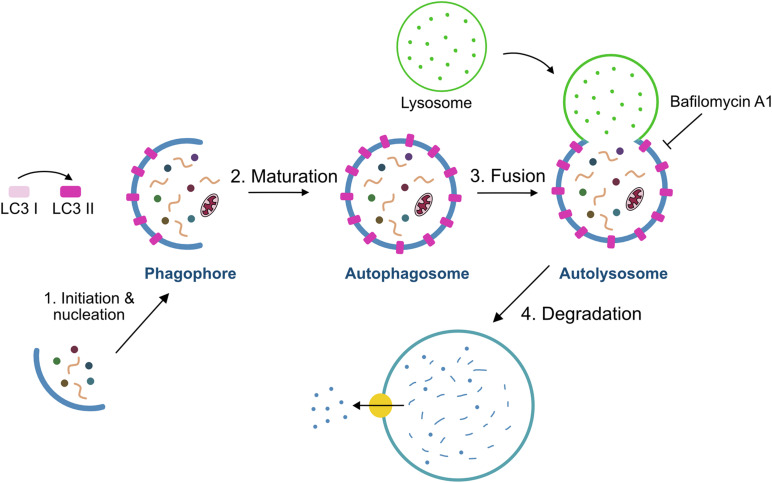
Schematic model of the autophagy process. The process of autophagy includes four steps: (i) initiation/nucleation where the phagophore is formed and starts engulfing the cargo, (ii) formation of the phagosome by elongation and maturation of the phagophore, (iii) fusion of the phagosome with the lysosome, and (iv) degradation of the internal material by lysosomal hydrolases. Bafilomycin A1 is used to measure autophagy flux through the inhibition of autophagolysosome formation.

The autophagy process is divided into sequential steps: initiation and nucleation, elongation, maturation, fusion and degradation (Figure 2). At the initiation of autophagy, a flat membrane sheet known as phagophore surrounds cytosolic components. This phagophore then elongates and seals itself forming a double-membrane bound vesicle called the autophagosome. Upon subsequent fusion with the lysosome, it gives rise to the autolysosome, whose intracellular components are rapidly degraded by the lysosomal hydrolases (Kroemer et al., 2010; Pyo et al., 2012). Several autophagy-related genes (Atg) products including the Atg8/Map1lc3b protein (microtubule-associated protein 1 light-chain 3, hereafter referred to as LC3) regulate autophagosome formation and maturation into autolysosomes. LC3 is a cytosolic protein that is cleaved and conjugated to phosphatidylethanolamine (PE) giving rise to the membrane-bound form of LC3, also referred to as LC3-II, the level of which is known to be correlated with the number of autophagosomes (Lee and Lee, 2016). Moreover, LC3-II in the autophagosome interacts with autophagy adaptors, such as p62/Sqstm1, Ndp52, Optn, Nbr1, or Tax1bp1, that act as a linkage between autophagosome and the substrate (Kirkin and Rogov, 2019). In sum, through the autophagy process, targeted cytoplasmic constituents are degraded in lysosomes.

Under metabolic stress, autophagy-mediated degradation of cytoplasmic constituents supports energy balance (Klionsky, 2005; Klionsky et al., 2010; Mizushima and Komatsu, 2011). Although autophagy was described in the beginning as a cellular process induced by stress, it also functions at baseline under quiescence in resting SCs, and this constitutive autophagic activity appears to be indispensable for maintaining stemness (García-Prat et al., 2016). Disruption of the autophagic capacity by Atg7 genetic deletion in young SCs leads to an increased accumulation of impaired mitochondria that cause high ROS levels, provoking further damage to proteins and DNA (García-Prat et al., 2016). Of interest, autophagy induction is also observed during SC activation *in vivo*, and blocking autophagy delays cell-cycle entry from the quiescent state, with consequences for muscle regeneration (Tang and Rando, 2014). Notably, the reported phenotype in autophagy-deficient murine SCs from young animals partly recapitulates the one observed in chronologically aged SCs (García-Prat et al., 2016). In fact, in contrast to young SCs, old SCs show defective autophagic activity. This autophagy failure ends up in a progressive accumulation of harmful intracellular waste, mainly composed of altered mitochondrial material, which produces high oxidative stress and DNA damage, leading to muscle stem cell senescence in very old (geriatric) mice (Sousa-Victor et al., 2014; García-Prat et al., 2016). Fiacco et al., recently showed that autophagy is induced during the early, compensatory regenerative stages of DMD. A gradual decline was observed throughout disease progression in dystrophic *mdx* mice, coinciding with the functional exhaustion of SC-mediated regeneration and accumulation of fibrosis. Furthermore, pharmacological modulation of autophagy could influence disease progression in *mdx* mutant mice. In support of this notion, interventions that prolong the activation of autophagy might be beneficial in treating DMD (Fiacco et al., 2016).

Because quiescent SCs exist in low numbers in resting muscles, are small in size, and have a low proportion of cytoplasm/nucleus (consistent with their quiescent state), the study of dynamic cytoplasmic processes, such as autophagy, in these cells is therefore challenging. Here, we show different methods to study autophagy in SCs, focusing on their quiescence state.

## Materials and Methods

### Mice

C57BL/6 (wild type, WT) and GFP-LC3 (Mizushima et al., 2004) mice were used in this study. All experiments were carried in young (3–4 months old) male mice. Mice were kept in standard cages with food and water *ad libitum*. All animals were sacrificed between 9:00 and 10:00 am by cervical dislocation to avoid circadian changes in autophagy (Solanas et al., 2017). All animal experiments were approved by the Ethics Committee of the Barcelona Biomedical Research Park (PRBB) and by the Catalan Government, by the Animal Care and Ethics Committee of the Spanish National Cardiovascular Research Center (CNIC) and, by the Regional Authorities.

### Satellite Cell Isolation by Fluorescence-Activated Cell Sorting

Muscles were collected from fore and hind limbs in cold DMEM (Gibco 41965-039) with 1% Penicillium/Streptomycin (P/S) (15140-122) into 50 mL Falcon tubes. Any visible fat and connective tissue were removed before mincing muscles with scissors. Cleaned and minced muscles were collected into a M tube (Miltelnyi Biotec, GentleMACS^TM^) and digestion medium (8 mL) was added. Digestion medium was freshly prepared with DMEM containing Liberase 0.1 mg/g muscle weight (Roche, 5401127001), Dispase 0.3% (Sigma-Aldrich, D4693-1G), 1% P/S, 0.4 μM CaCl_2_ and 5 μM MgCl_2_. M tubes were placed onto Miltelnyi tissue dissociator under the program 37C_mr_SMDK_1. Once it finished, tubes were kept 5 min on ice to sediment the sample and 5 mL of FBS (Sigma-Aldrich F7524) was added to block the enzymatic digestion. Next, digested muscles were transferred to a 50 mL Falcon tube and rinsed up to 40 mL with cold DMEM 1% P/S (Optional: cold DMEM can be used to rinse M tubes in order to collect leftovers and transferred to the same Falcon tube). Muscle homogenates were filtered through 100 and 70 μm cell strainers (SPL Lifescience, 93100 and 93070) consecutively and centrifuged at 50 × *g* for 10 min at 4°C. The supernatant was collected in a new Falcon tube and centrifuged at 600 × *g* for 10 min at 4°C. The supernatant was then discarded and the pellet was incubated for 10 min on ice (protected from light) with 1 mL of 1X RBC lysis buffer (eBioscience, 00-4333-57) to eliminate the excess of erythrocytes. To stop the lysis, 30 mL of 1X PBS was added and filtered through a 40 μm cell strainer (SPL Lifescience, 93040. Filtered cell suspension was centrifuged at 600 × *g* for 10 min at 4°C and after discarding the supernatant, the cell pellet was resuspended in 1 mL of DMEM with 1% P/S to count the number of cells for each sample.

For antibody staining, cell suspension was centrifuged at 600 × *g* for 10 min at 4°C and resuspended in cold FACS buffer (1% P/S, 5% Goat Serum (Gibco, 16210-064) in 1X PBS) containing antibody mixture at a ratio of 1 × 10^6^ cells/100 μL antibody mixture for 1 h at 4°C (protected from light). The antibody mixture contained antibodies for negative and positive selection of QSCs in FACS buffer. PE-Cy7-conjugated anti-CD45 (Biolegend 103114), anti-Sca-1 (Biolegend 108114) and anti-CD31 (Biolegend 102418) antibodies were used for lineage-negative selection at a ratio of 0.5 μL antibody/100 μL FACS buffer. Alexa Fluor 647-conjugated anti-CD34 (BD Pharmigen 560230) and PE-conjugated anti-α7-integrin (AbLab AB10STMW215) were used for double-positive staining of QSCs at a ratio of 3 μL/100 μL and 1 μL/100 μL FACS buffer respectively. Optionally, single staining and FMO controls can be included to set up correctly the gates. After staining, samples were rinsed up to 30 mL of FACS buffer and centrifuged at 600 × *g* for 10 min at 4°C to wash the excess of antibodies. Samples were then resuspended in 300 μL FACS buffer with DAPI (1 μg/mL) (Invitrogen, D1306) to exclude dead cells. Finally, Sca1^–^/CD31^–^/CD45^–^/CD34^+^/α7-integrin^+^ SCs were collected into Eppendorf tubes containing 100 μL of collection medium (Ham’s F10 (Biowest L0140-500), 1% P/S, 1% Glutamine (Lonza, 17-605E), 20% FBS) at 4°C using a FACS Aria II (BD Biosciences).

### Drug Treatment for Autophagy Flux Determination in Quiescent Satellite Cells

Prior to antibody staining and quiescent SC isolation by FACS, samples (already resuspended in 1 mL of DMEM with 1% P/S) were centrifuged at 600 × *g* for 10 min at 4°C. The cell pellet was resuspended in 1 mL of collection medium. Each sample was split into two new 2 mL Eppendorf tubes for Bafilomycin A1 (10 nM; Sigma, B1793) or DMSO (vehicle; Sigma, D2540) treatment for 4 h at 37°C 5% CO_2_. After drug treatment, antibody staining for SC isolation was performed as mentioned above.

### Drug Treatment for Autophagic Flux Determination in Activated and Proliferating Satellite Cells

Freshly sorted SCs were cultured on 15-well plastic slides (μ-Slide Angiogenesis ibiTreat: Ibidi, 81506) previously coated with collagen type I (Corning, 354236) in growth medium [GM; collection medium supplemented with recombinant bFGF (Preprotech, 100-18B, 0.0025 μg/mL)]. A total number of 3000 SCs per well were homogenously distributed plated. After 20 or 68 h in culture, cells were treated with Bafilomycin A1 at 10 nM or DMSO during the last 4 h for activation and proliferation SCs states. Cells were then fixed at 24 and 72 h respectively.

### Flow Cytometry Analysis of Autophagy in Quiescent Satellite Cells

Using GFP-LC3 reporter mice, GFP fluorescence signal was recorded from at least 10.000 Sca1^–^/CD31^–^/CD45^–^/CD34^+^/α7-integrin^+^ SCs in each sample. GFP-LC3 fluorescence positive signal was determined comparing to the corresponding negative control sample (wild-type sample without GFP fluorescence). Median fluorescence intensity (MFI) of the whole GFP histogram signal for SCs was analyzed. Autophagy flux was determined as the relative change of GFP-LC3 MFI between DMSO and Bafilomycin A1 treated samples.

### Immunofluorescence and Image Acquisition of Satellite Cells

Isolated quiescent SCs were plated onto 15-well plastic slides (μ-Slide Angiogenesis ibiTreat: Ibidi, 81506). Prior to SC plating, slides were coated with 0.1% Poli-L-Lysin (Sigma, P8920) in distilled water (it can be reused) for 30 min at room temperature (RT) and air-dried. For each sample, 3000 SCs per well were seeded into the 15-well slides. Eventually, 1X PBS can be added to the wells in order to ensure that the cell suspension is equally distributed. To cytospin the cells, the slides were then centrifuged at 50 × *g* for 10 min.

For both cultured and quiescent SCs, the supernatant was removed and cells were fixed with 30 μL of 4% PFA for 10 min at RT. After fixation, two washes with 1X PBS were performed. At this time point, slides can be stored with 1X PBS 0.05% azide at 4°C. It is recommended to fill completely each well and cover them with parafilm to avoid PBS evaporation.

After fixation, slides were permeabilized with 0.5% Triton X-100 (Sigma, T8787) for 15 min at RT and washed three times with 1X PBS. Next, wells were incubated 30 min at RT with blocking solution containing BSA (Sigma, A7906, 3 mg/mL) in 1X PBS. Primary antibodies (Table 1) were diluted in blocking solution and incubated for 2 h at RT or overnight (O/N) at 4°C. Antibodies were removed and wells were washed three times with 1X PBS. Slides were incubated with secondary antibody solution for 1 h at RT (Table 1). DAPI (1 μg/mL) or Sytox^TM^ Green (Invitrogen, S7020, 1/15000) were used for nuclear staining. Each well was extensively washed three times with 1X PBS and finally Fluoromount-G^®^ (SouthernBiotech, 0100-01) mounting media was added. Optionally, a drop of mineral oil can be added to the top of the well for long-term storage.

**TABLE 1:** List of antibodies used in this article.

Antibody	Company	Reference	Source	Dilution
Anti-CD45 PE-Cy7	Biolegend	103114	Rat	0.5/100*
Anti-Sca-1 PE-Cy7	Biolegend	108114	Rat	0.5/100*
Anti-CD31 PE-Cy7	Biolegend	102418	Rat	0.5/100*
Anti-CD34 Alexa Fluor-647	BD Pharmigen	560230	Rat	3/100*
Anti- α7-integrin PE	AbLab	AB10STMW215	Rat	1/100*
Anti-GFP	Aves labs	GFP-1020	Chicken	1/200
Anti-LC3	Nanotools	5F10	Mouse	1/100
Anti-MyoD	Dako	M3512	Mouse	1/200
Anti-Ki67	Abcam	ab15580	Rabbit	1/200
Anti-Chicken FITC	Aves Labs	F-1005	Goat	1/500
Anti-Chicken Alexa Fluor-405	Life Technologies	Ab175674	Goat	1/500
Anti-Mouse Alexa Fluor-647	Life Technologies	A-31571	Donkey	1/500
Anti-Rabbit Alexa Fluor-647	Life Technologies	A-21245	Donkey	1/500

*SCs were previously diluted in a ratio of 1 × 10^6^ cells/100 μL.

### Image Acquisition and Analysis of Autophagy in Satellite Cells

Digital images were acquired using a Zeiss LSM 700 confocal microscope with a Plan-Apochromat 63x/1.4 NA oil objective. At least 25–30 SCs per sample were imaged using confocal z-stack (0.5 μm interval). Zeiss LSM software Zen Black was used for digital acquisition and Fiji software was used for further image processing. As image preprocessing, gaussian smoothing (radius = 0.4) and background subtraction (ball radius = 20) were applied to the whole z-stack. Then, autophagosomes per cell defined as GFP-LC3^+^ puncta were identified as individual 3D objects using the 3D Roi Manager plugin (Ollion et al., 2013). Whenever possible, measurements were performed blindly.

### Statistical Analysis

GraphPad Prism (GraphPad Software, Inc) software was used for all statistical analysis. Data are presented as the mean ± the standard deviation of the mean. Sample size (n) of each experimental group was described in the corresponding figure legend and all experiments were done with at least three biological replicates. Normality was analyzed in each experiment using Shapiro-Wilk tests and homoscedasticity to test variances distribution was checked using the Fisher test. For normally distributed data, two-tail unpaired Student’s t-test was performed. Statistical significance was set at ^∗^*p* < 0.05, ^∗∗^*p* < 0.01, ^∗∗∗^*p* < 0.001, ^****^p < 0.0001.

## Results and Discussion

### Satellite Cell Isolation by FACS

**FIGURE 3:**
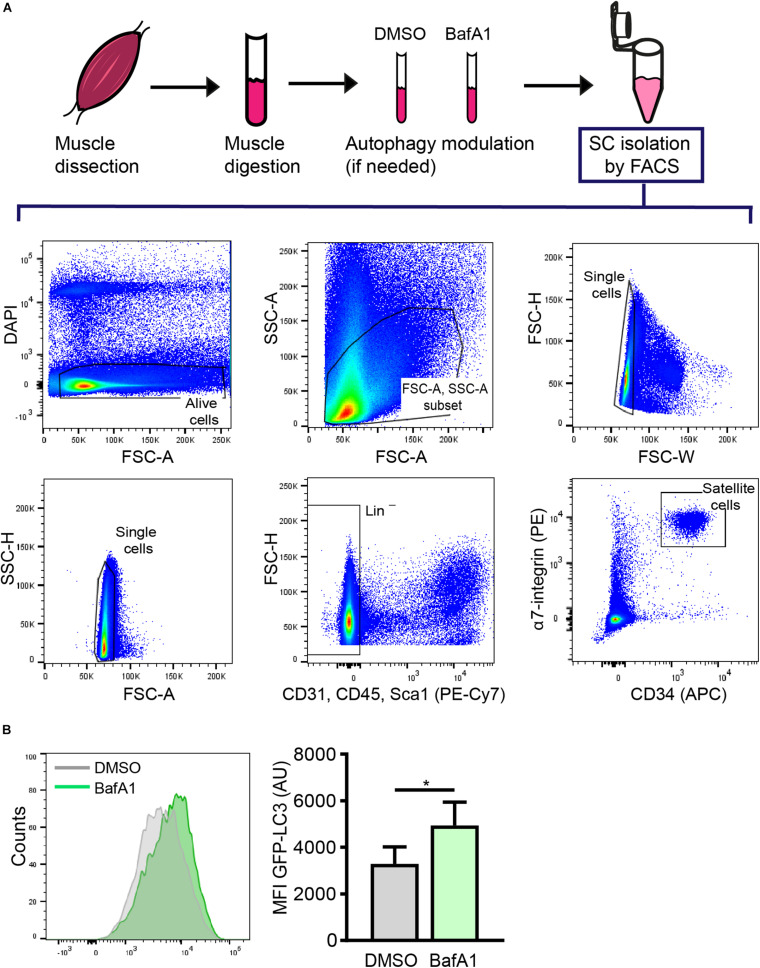
Satellite cell isolation by FACS and subsequent analysis of autophagy through flow cytometry. **(A)** Representative example of the FACS strategy and gating scheme to isolate quiescent SCs (QSCs) rom resting muscles. **(B)** Representative example of histogram of LC3-GFP intensity (left panel) and analysis of the mean fluorescence intensity (MFI) by flow cytometry (right panel) in QSCs treated for 4 h with vehicle (DMSO) or BafA1 prior to their isolation by FACS (*n* = 4). Mean ± SD; two-tailed unpaired *t*-test. *p < 0.05.

The proper isolation of SCs is crucial for characterizing the mechanisms involved in stem-cell quiescence maintenance and/or regenerative functions. Several methods based on fluorescence-activated cell sorting (FACS) using different cell surface markers for positive and negative cell selection have been optimized to isolate SCs (Sherwood et al., 2004; Joe et al., 2010; Pasut et al., 2012; Liu et al., 2013). Here, we used a FACS protocol based on CD45/CD31/Sca1 negative (Lin^–^) cells and μ7-integrin/CD34 double positive cells (Lin^–^/μ7-integrin^+^/CD34^+^) to isolate quiescent SCs from resting muscle (Figure 3A).

### Autophagy Flux Determination in Quiescent Satellite Cells

As autophagy is a multistep process (see Figure 2), identification and quantification of autophagosomes within a cell at a given time-point is insufficient to report this dynamic process. Instead, the balance between the rate of autophagosome generation and its incorporation into autolysosomes (i.e., autophagic flux) is the optimal way to assess autophagy (Mizushima et al., 2010). Autophagy flux assays use inhibitors of autophagosome incorporation into the lysosome to discern between autophagosome formation and clearance. We have used Bafilomycin A1 (BafA1), a vacuolar H^+^-ATPase inhibitor that blocks vesicle acidification as well as fusion between autophagosomes and lysosomes (Mauvezin et al., 2015).

The autophagy flux can be assessed by measuring relative levels of p62 or measuring the ratio between the lipidated form of LC3 (LC3-II) and the unconjugated form (LC3-I) (Kabeya, 2004) from protein extracts (granted that enough material is available to perform standard Western blotting). Unfortunately, the low numbers of freshly isolated SCs per mg of muscle tissue and their reduced cytoplasmic content, and therefore, low protein content, make this option unfeasible unless the starting material is scaled up (pooling tissue from several mice per experiment).

**FIGURE 4:**
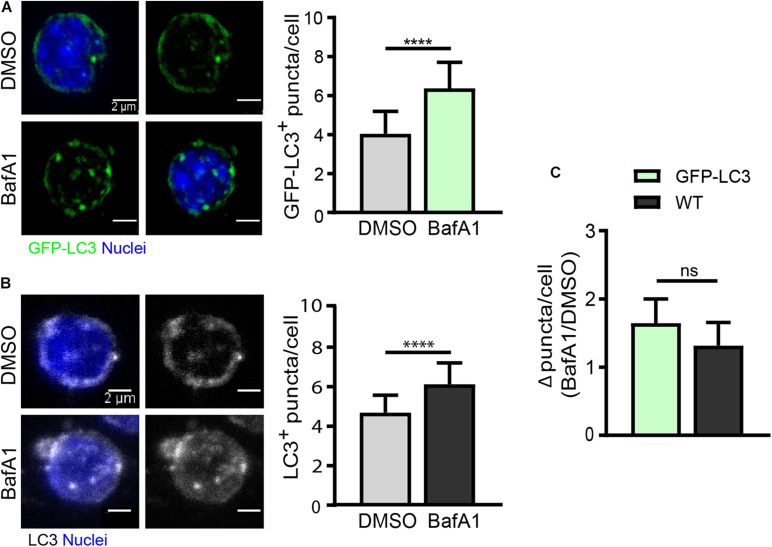
Autophagy flux analysis in quiescent satellite cells by immunofluorescence. **(A)** Representative images of GFP^+^-autophagosomes (green) and nuclei (blue) in freshly isolated QSCs from GFP-LC3 reporter mice (left panel) with the corresponding quantification of GFP-LC3^+^ puncta per cell (right panel). Treatment with vehicle (DMSO) or BafA1 was performed for 4 h prior QSC isolation by FACS (*n* = 4). Scale bar, 2 μm. **(B)** Representative images of LC3-stained autophagosomes and nuclei (blue) in freshly isolated QSCs from WT mice (left panel) with its corresponding quantification of LC3^+^ puncta per cell (right panel). Treatment with vehicle (DMSO) or BafA1 was performed for 4 h prior QSC isolation by FACS (*n* = 3). Scale bar, 2 μm. **(C)** Comparison of autophagy flux in GFP-LC3 or WT QSCs. Autophagy flux was determined as the ratio of the number of autophagosome puncta in BafA1 treated QSCs divided by the number of autophagosome puncta in vehicle (DMSO) treated QSCs (for GFP-LC3: *n* = 4; for WT: *n* = 3). Means ± SD; two-tailed unpaired *t*-test. ****p < 0.0001.

As quiescent SCs have a low cytoplasmic content, the identification of autophagosomes within the cytoplasm is a challenging task. We have set up an autophagy flux protocol in SCs isolated from a GFP-LC3 reporter mouse line, in which autophagosomes are labeled with GFP (green florescent protein). Although freshly isolated SCs from steady-state skeletal muscle are considered quiescent cells, one important point to consider when studying the quiescent state *ex vivo* is the potential changes induced in freshly isolated cells during the tissue’s mechano-enzymatic disruption and subsequent isolation procedures, particularly at the transcriptional level (Machado et al., 2017; van Velthoven et al., 2017). Digested muscle was treated with BafA1 or vehicle (DMSO) for 4 h prior to quiescent SC isolation by FACS (see “Drug Treatment for Autophagy Flux Determination in Quiescent Satellite Cells” and scheme in Figure 3A for further details). After SC isolation, the autophagy flux in quiescent SCs was determined by monitoring the GFP-LC3 fluorescence levels by flow cytometry. We observed an increase in the GFP-LC3 intensity upon BafA1 treatment (Figure 3B). To assess whether this increase is due to an accumulation of autophagosomes, we quantified the number of GFP^+^-autophagosomes in BafA1-treated quiescent SCs by immunofluorescence, and found that the number of GFP^+^-autophagosomes was increased in BafA1-treated compared to vehicle-treated quiescent SCs (Figure 4A).

As GFP-LC3 reporter mouse strains may not be always available, we determined the autophagy flux in quiescent SCs isolated from WT mice by immunostaining the endogenous LC3 in cells treated or not with BafA1 (Figure 4B). As for GFP-LC3 reporter SCs, the number of autophagosomes was increased in BafA1-treated compared to vehicle-treated quiescent SCs (Figure 4C); moreover, similar autophagy flux ratios were found in GFP-LC3 reporter and WT quiescent SCs (Figure 4C) despite the higher background observed in endogenous LC3-staining conditions. Of note, it should be feasible to combine the GFP-LC3 fluorescence or the endogenous LC3 staining with other autophagy markers such as p62 (adaptor protein) and ubiquitin (in the cargo) aggregates to assess their potential colocalization. Since p62 is a marker of damaged organelles to be eliminated by autophagy and ubiquitin marks substrates for elimination by autophagy or the ubiquitin-proteasome system (UPS) (reviewed in Liu et al., 2016), the colocalization of LC3 with these markers may serve to further assess autophagy defects or autophagy flux impairments. Other lysosomal inhibitors can also be used for the assessment of autophagy flux, including lysosomal lumen “alkalizers” such as chloroquine or NH4Cl, as well as acid protease inhibitors such as leupeptin (Yang et al., 2013).

### Autophagy Flux Determination in Activated and Proliferating Satellite Cells

**FIGURE 5:**
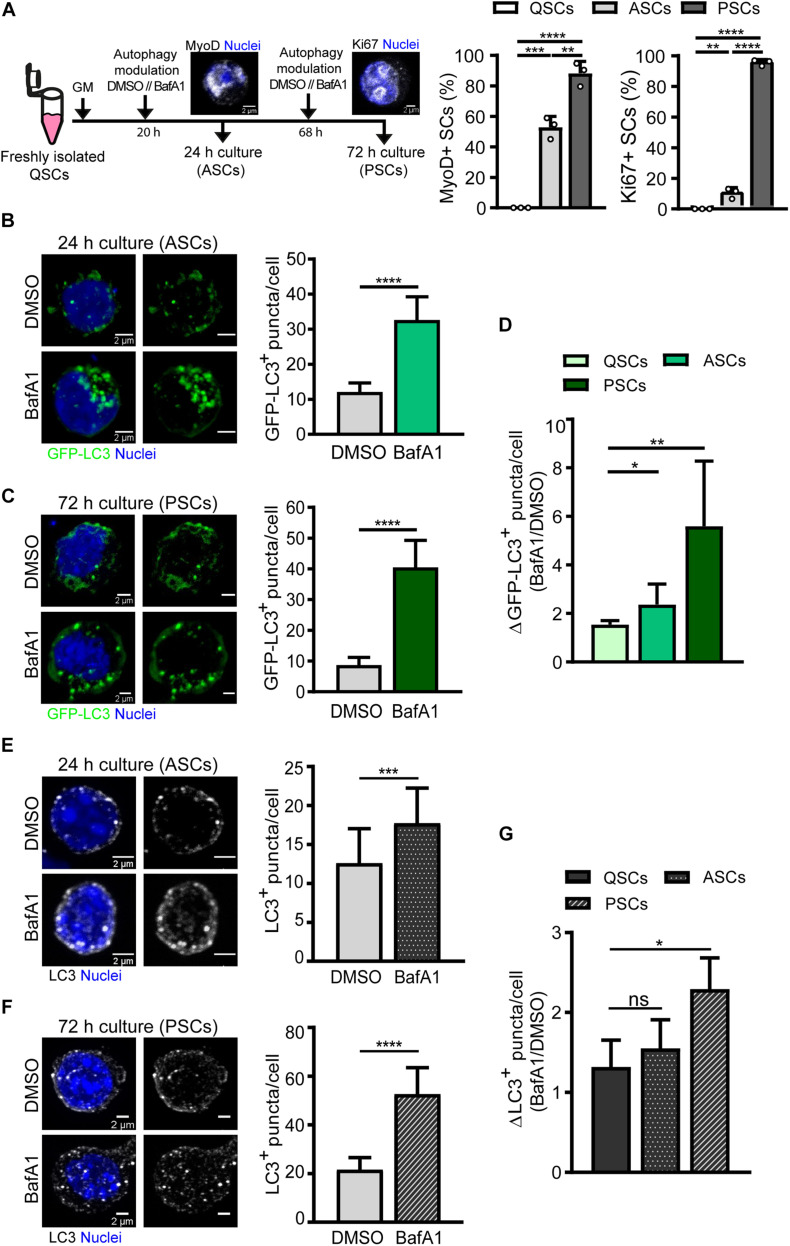
Autophagy flux analysis in activated and proliferating satellite cells by immunofluorescence. **(A)** Right panel: Scheme of the process followed for autophagy flux assessment in (1) activated SCs (ASCs), characterized by the presence of MyoD protein and still lacking cell-cycle proteins such as Ki67, and (2) proliferating SCs (PSCs), marked by the expression of the proliferative marker Ki67. Left panels: quantification of the percentage of MyoD^+^ and Ki67^+^ cultured SCs at 24 and 72 h time points. **(B,C)** Representative images of GFP^+^-autophagosomes (green) and nuclei (blue) in ASCs **(B)** and PSCs **(C)** from GFP-LC3 reporter mice (left panels) with the corresponding quantification of GFP-LC3^+^ puncta per cell (right panels). Treatment with vehicle (DMSO) or BafA1 was performed for 4 h prior fixation (*n* = 3). Scale bar, 2 μm. **(D)** Autophagy flux represented as the ratio of GFP-LC3^+^ puncta in BafA1 and vehicle (DMSO) along SC myogenesis *in vitro* (*n* = 3–6). **(E,F)** Representative images of LC3 + -autophagosomes (gray scale) and nuclei (blue) in ASCs **(E)** and PSCs **(F)** from WT mice (left panels) with the corresponding quantification of LC3^+^ puncta per cell (right panels). Treatment with vehicle (DMSO) or BafA1 was performed as in panels **(B,C)** (*n* = 4). Scale bar, 2 μm. **(G)** Autophagy flux, depicted as the ratio of LC3^+^ puncta in BafA1 and vehicle (DMSO), along SC myogenesis *in vitro* (*n* = 4). Means ± SD; two-tail unpaired *t*-test. *p < 0.05, **p < 0.01, ***p < 0.001, and ****p < 0.0001.

We next analyzed the differences in autophagy flux in activated and proliferating SCs compared to quiescent cells. Freshly FACS-isolated SCs carrying the GFP-LC3 reporter were cultured for 24 and 72 h, corresponding to activation and proliferation states, respectively (Figure 5A). At 24 h, the cell cycle marker Ki67 is only expressed in around 10% of SCs, indicating that most SCs have not yet achieved the full proliferation state at this time point. Concurrently, SCs start to express the myogenic regulatory factor MyoD (Figure 5A). In contrast, at 72 h, most SCs (95%) are actively proliferating and become immunopositive for Ki67 (Figure 5A). Cells were treated with vehicle or BafA1 for 4 h prior to fixation and autophagy flux was measured by counting GFP-LC3 autophagosomes in activated SCs (MyoD^+^ cells, after 24 h culture) and in proliferating SCs (Ki67^+^ cells, after 72 h culture) (Figures 5B,C). Autophagy flux was estimated as the difference between autophagosome formation and degradation at a given time-window. Since SCs differ in their cellular size along the distinct myogenic stages, the number of autophagosomes upon BafA1 treatment was divided by the number of autophagosomes upon DMSO treatment for autophagy flux normalization, thus reducing cell size-induced variability. We found that autophagy flux was increased upon SC activation from quiescence, and this increase was even higher at the proliferation stage (Figure 5D).

Autophagy flux was also analyzed in WT freshly FACS-isolated SCs cultured for 24 and 72 h following the approach described in Figure 5A. Immunostaining of endogenous LC3 was performed for autophagosome detection and autophagy flux measurement in SCs treated with vehicle or BafA1, showing an increase in the number of autophagosomes in BafA1-treated SCs (Figures 5E,F). Moreover, similar to what is observed in transgenic SCs expressing the GFP-LC3 reporter, the highest autophagy flux was found at the proliferation stage (Figures 5D,G). However, although WT SCs showed a trend to increase their autophagy flux upon activation, we did not obtain significant differences in activated SCs with respect to their quiescent state (Figure 5G). Of note, the ratios for autophagy flux during activation and proliferation were smaller in WT SCs compared to transgenic SCs (Figures 5D,G).

GFP-LC3 can be incorporated into protein aggregates (Hara et al., 2006; Komatsu et al., 2006), and given that protein synthesis (and probably protein aggregates) increases upon cell division, the transgene would be more prone to be incorporated into these aggregates in activated and proliferating SCs, mainly if the reporter’s cellular levels are high. This possibility could explain why the differences in autophagy flux ratios in WT and transgenic SCs are higher upon SC cell cycle entry while remaining more similar at quiescence. Moreover, the distinct background observed with endogenous LC3 staining may also influence autophagosome detection and subsequent ratio determinations.

In summary, despite the slight differences in determining autophagy flux with both approaches, the autophagy activity increases upon SC activation and consequent proliferation. Therefore, the use of either GFP-LC3 reporter or endogenous LC3 staining provides an easy and robust way to analyze autophagy during SC myogenesis *in vitro*. Indeed, these results are in agreement with previous observations demonstrating a role for autophagy in quiescence maintenance and in supporting cell survival and metabolic demands upon stem-cell exit from quiescence and entrance into proliferation to ensure successful muscle regeneration (Tang and Rando, 2014; García-Prat et al., 2016).

## Conclusion

The small proportion of SCs present in steady-state skeletal muscle and their reduced cytoplasm in the quiescent state after sorting, challenges the monitoring of autophagy in these cells. The protocols reported here enable quiescent SC isolation by FACS from resting murine skeletal muscle tissue using standard laboratory equipment and allows us to study their autophagy activity at distinct myogenic stages. We describe different experimental strategies for autophagy flux determination by either flow cytometry or immunofluorescence in quiescent SCs and their activated and proliferating progeny. These methods provide the scientific community with useful approaches for assessing autophagy in scenarios in which autophagy is genetically altered or in aging and disease conditions.

## Data Availability Statement

The raw data supporting the conclusions of this article will be made available by the authors, without undue reservation.

## Ethics Statement

This study was carried out in accordance with the EU Directive 86/609/EEC and approved by the National and Regional Animal Care and Ethics Committees.

## Author Contributions

SC and IR-P performed the experimental work. SC, IR-P, and PM-C wrote the manuscript. SC, IR-P, and XH assembled the figures. SC, JI, and PM-C supervised the manuscript. PM-C approved the final version of the manuscript. All the authors contributed to the data analysis and interpretation and discussed the results.

## Conflict of Interest

The authors declare that the research was conducted in the absence of any commercial or financial relationships that could be construed as a potential conflict of interest.
